# Development and Measurements of a Mid-Infrared Multi-Gas Sensor System for CO, CO_2_ and CH_4_ Detection

**DOI:** 10.3390/s17102221

**Published:** 2017-09-27

**Authors:** Ming Dong, Chuantao Zheng, Shuzhuo Miao, Yu Zhang, Qiaoling Du, Yiding Wang, Frank K. Tittel

**Affiliations:** 1 State Key Laboratory on Integrated Optoelectronics, College of Electronic Science and Engineering, Jilin University, Changchun 130012, China; 1988dm@sina.com (M.D.); miaosz16@163.com (S.M.); yuzhang@jlu.edu.cn (Y.Z.); duql@jlu.edu.cn (Q.D.); ydwang@jlu.edu.cn (Y.W.); 2 Electrical and Computer Engineering Department, Rice University, 6100 Main Street, Houston, TX 77005, USA; fkt@rice.edu

**Keywords:** mid-infrared absorption spectroscopy, multi-gas detection, gas sensor

## Abstract

A multi-gas sensor system was developed that uses a single broadband light source and multiple carbon monoxide (CO), carbon dioxide (CO_2_) and methane (CH_4_) pyroelectric detectors by use of the time division multiplexing (TDM) technique. A stepper motor-based rotating system and a single-reflection spherical optical mirror were designed and adopted to realize and enhance multi-gas detection. Detailed measurements under static detection mode (without rotation) and dynamic mode (with rotation) were performed to study the performance of the sensor system for the three gas species. Effects of the motor rotating period on sensor performances were also investigated and a rotation speed of 0.4π rad/s was required to obtain a stable sensing performance, corresponding to a detection period of ~10 s to realize one round of detection. Based on an Allan deviation analysis, the 1*σ* detection limits under static operation are 2.96, 4.54 and 2.84 parts per million in volume (ppmv) for CO, CO_2_ and CH_4_, respectively and the 1*σ* detection limits under dynamic operations are 8.83, 8.69 and 10.29 ppmv for the three gas species, respectively. The reported sensor has potential applications in various fields requiring CO, CO_2_ and CH_4_ detection such as in coal mines.

## 1. Introduction

The development of multi-gas detection technology plays an important role in many areas, such as environmental atmospheric monitoring [1,2], medical diagnosis [3,4], industrial process control [5,6], gas monitoring and fire alarm systems in coal mines [7,8,9,10]. So far, the most widely used technology for multi-gas instruments is photo-acoustic spectroscopy (PAS) and catalytic reaction. PAS detects gas concentration by acquiring acoustic signals in the gas chamber based on the photoacoustic principle [11,12,13]. However, environment vibrations will interfere with normal sensor operation and cause measurement errors of the PAS sensor. Additionally, a multi-gas sensor based on a catalytic reaction has a much shorter service lifetime compared to infrared (IR) sensors [14,15,16]. Direct absorption spectroscopy (DAS), however, has the advantage of a compact design [17]. In addition, a broadband thermal source and pyroelectric detector in a DAS technique are less costly compared to a mid-infrared laser and detector. 

There are many ways to realize a multi-gas detector, which can be divided into three types: multiple light sources with a single detector, single broadband light source with multiple detectors, and a single tunable light source with a single detector. For example, Besson [18] demonstrated a multi-gas sensor using three near-infrared distributed feedback lasers (DFB) and a resonant photoacoustic cell. Mukherjee [19] designed a non-dispersive optical multiplexer using a scanning galvanometer for multispecies trace-gas detection, where five quantum cascade lasers (QCLs) were used, using time division multiplexing (TDM). Tan [20] integrated four single-channel pyroelectric sensors in a miniature optical gas chamber based on a non-dispersive infrared (NDIR) technique. Mao [21] reported an all-optical photoacoustic spectrometer for multi-gas analysis based on a tunable erbium-doped fiber laser and a fiber acoustic sensor. As the emission spectrum of a single laser cannot cover a wide wavelength band, multiple lasers have to be used to achieve multi-gas detection. The lasers used in tunable diode laser absorption spectroscopy (TDLAS) and PAS techniques are generally costlier than an infrared thermal source or light emitting diode (LED).

The developed sensor is intended to be used in the mining industry, where the normal CO, CO_2_ and CH_4_ concentration are less than several ppm, hundreds to thousands of ppm and thousands of ppm, respectively. So, the detection limits of CO, CO_2_ and CH_4_ are designed to be <10 ppm, <100 ppm and <100 ppm, respectively, to meet the requirements of mining detection. 

In order to reduce sensor cost, a single broadband light source and multiple detectors were used in this work to realize a multi-gas sensor based on DAS. The emission spectrum of the wideband light source covers the mid-infrared absorption band of CO (4.65 µm), CO_2_ (4.26 µm), CH_4_ (3.31 µm), which are the fundamental absorption bands of these three gas species. A mechanical rotation module was designed to switch between the three gas detectors and three gases were detected in sequence using the time division multiplexing (TDM) technique. Both static (without rotation) and dynamic (with rotation) detection characteristics were studied. The structure of this paper is organized as follows: the sensor structure will first be described followed by detection theory, optical and mechanical design, and the closed-loop control theory of the mechanical rotation module; sensor validation as well as measurement results of the detection limit, response time and stability will then be proposed. Finally, a discussion and conclusion section will be presented.

## 2. Multi-Gas Sensor Structure and Design

### 2.1. Sensor Structure

**Figure 1:**
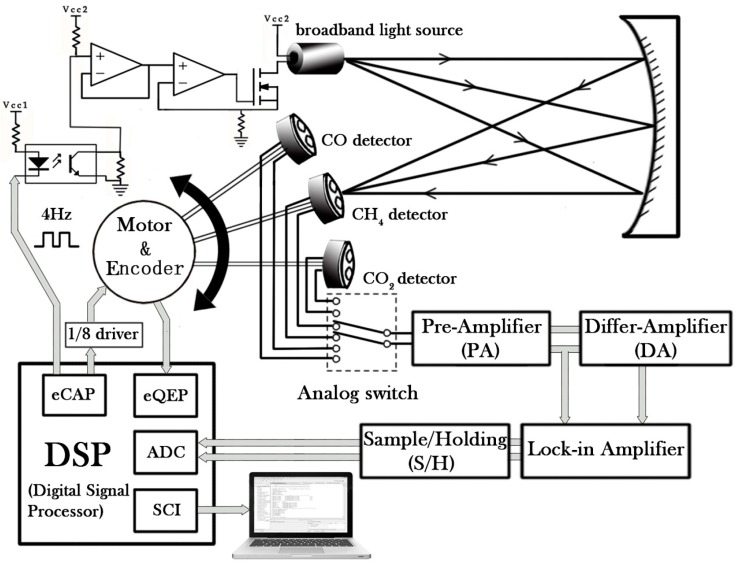
Schematic of the multi-gas sensor system using a broadband light source and a three dual-channel pyroelectric detector system.

As shown in Figure 1, the proposed multi-gas sensor system can be divided into optical, mechanical and electrical parts. In the optical part, the light emitted by a broadband light source (IR-55, Boston Electronics, Brookline, MA, USA) is reflected by a spherical mirror with a focal length of 100 mm and converges at a pyroelectric detector. The detector has a reference channel and a detection channel with two different filters installed in the window assembly. The detector generates a detection signal and a reference signal. The three dual-channel pyroelectric detectors (Infra Tec GmbH, Dresden, Germany) with the different wavelengths for CO, CO_2_, CH_4_ sensing are mounted on a disc with an interval angle of 45°. The disc is driven by a stepper motor. When a detector moves to a focal point, it will remain at this point for the time required for gas detection. 

The sensor system switches the three detectors continuously to detect the three gases, which can then be processed by the TDM technique. In order to improve the accuracy and stability of the rotation system, a 1/8 subdivided motor driver and an incremental encoder were used for feedback of the current angle of the stepper motor to form a closed-loop rotation control system. In the electrical part, a digital signal processor (DSP) reads the current angle from the encoder by means of an enhanced quadrature encoder pulse module (eQEP). 

A closed-loop algorithm was used to precisely control the rotation and switching of the three detectors. A 4-Hz square-wave signal generated by the DSP by means of an enhanced capture module (eCAP) was used to modulate the broadband light source through a constant current circuit. An analog switch was then used to switch the sensing channels of the three detectors. When a detector starts to operate, it will generate both a detection signal *u*_1_(*λ*) and a reference signal *u*_2_(*λ*). These two signals are first subjected to an impedance match by a preamplifier (PA), which is followed by a differential operation (DA) for generating a differential signal, i.e., Δ*u*(*t*) = *u*_2_(*t*) − *u*_1_(*t*). The amplitudes of *u*_2_(*t*) and Δ*u*(*t*) are then extracted by a custom-made lock-in amplifier. The light source modulation signal is used as a reference signal for the lock-in amplifier. The lock-in amplifier will produce a square wave which has the same frequency as the reference signal. 

The square wave is phase-shifted with respect to the reference signal by the DSP. The signal to be measured and the phase-shifted signal are then multiplied by an analog multiplier after passing through a sampling/holding circuit (S/H) and the output of the multiplier is then fed back to the DSP for sampling. The DSP adjusts the phase difference between the reference and the phase-shift signals according to the feedback and the optimum phase shift obtained with a maximized feedback signal. The output of the lock-in amplifier varies linearly with the amplitude of the sensing signal to be measured. The maximized feedback signal is processed with a sliding average filtering algorithm and finally the results are transmitted to a laptop for real-time monitoring.

### 2.2. Detection Theory and Consideration

The Beer–Lambert law in Equation (1) shows the relationship between gas concentration *C* and the received light intensity *I*, as

(1) $$I(\lambda )=I_{0}(\lambda )e^{-K(\lambda )CL}$$

where *I*_0_ is emission intensity, *K* is a molecule absorption coefficient and *L* is optical path length. With modulation, the time-dependent light intensity *I*(*λ*_1_) at *λ*_1_ = 4.65, 4.26, 3.31 µm (corresponding to CO, CO_2_, CH_4_) and the time-dependent light intensity *I*(*λ*_2_) at *λ*_2_ = 3.95 µm will be converted to two voltage signals, whose amplitudes are *U*_1_ and *U*_2_, respectively. The final gas concentration can be expressed by Equation (2)

(2) $$C=-\frac{1}{KL}\ln(1-\frac{\Delta U}{U_{2}})$$

where Δ*U* is a differential signal, i.e., Δ*U* = *U*_1_ − *U*_2_. The signal-to-noise-ratio (SNR) of the sensor system can be improved by differential operation. Moreover, the light intensity change caused by interference of dust on the mirrors and source current variations will result in changes of Δ*U* and *U*_2_ in a similar way, so that the ratio of Δ*U*/*U*_2_ can be helpful in reducing the impact of interferences.

Since the selected wavelength bands (3.31 µm for CH_4_, 4.25 µm for CO_2_, 4.65 µm for CO) for the three gases are fundamental bands, there is no interference among the three target gas species. So, during gas detection, the main interference may come from H_2_O, since H_2_O has a lot of absorption lines within the infrared wavelength band. 

The developed sensor is intended to be used in CH_4_ detection in the mining industry, where the normal CH_4_ concentration is several thousands of ppm. With reference to the high-resolution transmission (HITRAN) molecular absorption database, one can get the detailed absorption spectra of the 1000 ppm CH_4_ and the 2% H_2_O (relative humidity is ~80% at 25 °C) within the spectral range of the two filter windows of the dual-channel CH_4_ detector (one filter was centered at 3.31 µm with a half pass bandwidth of 167 nm, another filter was centered at 3.957 µm with a half pass bandwidth of 91 nm). The 3.31 µm window covers the strong absorption lines of CH_4_, which are helpful for enhancing sensitivity. The CH_4_ absorption within the range of 3.9–4.0 μm is at least three times smaller than the absorption at 3.31 μm. So, the 3.95-μm channel can be used as a reference channel for background elimination. Under 1 atm and 298 K, the absorption of H_2_O is at least 2–3 orders smaller than CH_4_ absorption within the two sensing windows, so it has almost no effect on CH_4_ detection. 

The normal CO_2_ concentration is around several hundreds of ppm in the mining industry. With reference to the HITRAN database, one can get the detailed absorption spectra of the 300 ppm CO_2_ and the 2% H_2_O within the spectral range of the two filter windows of the dual-channel CO_2_ detector (one filter was centered at 4.26 µm with a half pass bandwidth of 198 nm, another filter was centered at 3.957 µm with a half pass bandwidth of 91 nm). The absorption of H_2_O is at least 7 orders smaller than CO_2_ absorption within the two sensing windows, so it has almost no effect on CO_2_ detection. 

The CO level, on the other hand, is ~10 ppm in the mining industry. Based on the detailed absorption spectra of the 10 ppm CO and the 2% H_2_O within the spectral range of the two filter windows of the dual-channel CO detector (one filter was centered at 4.65 µm with a half pass bandwidth of 175 nm, another filter was centered at 3.957 µm with a half pass bandwidth of 91 nm), the absorption of H_2_O is a bit smaller than CO absorption within the two sensing windows. So, some measures, e.g., using driers, should be adopted to dry the air before entering the gas cell. 

### 2.3. Optical and Mechanical Design

**Figure 2:**
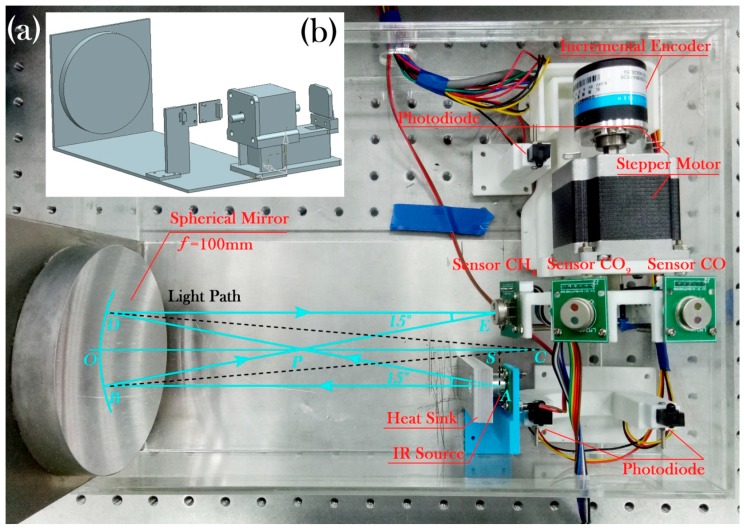
(**a**) Photograph of the optical part of the multi-gas sensor; (**b**) Computer Aided Design (CAD) model of the optical support frame.

Figure 2a shows a photograph of the optical part of the multi-gas sensor, where the blue line indicates the light path. Figure 2b shows the Autodesk Computer Aided Design (CAD) model of the optical support frame. A software tool named unigraphics (UG) was used for the CAD design. The support frame and the disc were fabricated using 3-D printing technology. 

As shown in Figure 2a, one of the outermost rays (*AB*) from the light source (*A*) is parallel to the axis *OC* and the reflected light (*BE*) will pass through the focus (*P*). Next, the other outermost ray (*AD*) from the light source passes through the focus (*P*) and the reflected light (*DE*) will be parallel to the axis *OC*, *BE* and *DE* intersect at point *E* at which the detector is rotated. Points *A* and *E* are symmetrical about the axis *OC*. The focal length of the spherical mirror is 100 mm and the specific position parameters can be calculated by the following formula:

(3) $$\left\{ \begin{array}{l} SP=AP\cdot \cos∠BAP=f\cdot \cos15{}^{\circ}=96.6\\ SO=SP+PO=96.6+100=196.6\\ AS=AP\cdot \sin∠APC=f\cdot \sin15{}^{\circ}=25.9 \end{array}\right.$$

Finally, we obtain the coordinate of point *A* (196.6, −25.9), and the coordinate of point *E* (196.6, 25.9) relative to point *O* (0, 0), which is the design parameters of the light path. The optical path length is calculated to be 393.2 mm, i.e., twice the length of line *SO*.

The disc is fixed on the front axle of the stepper motor and an incremental encoder is attached on the rear axle of the stepper motor to monitor the rotation. Two pairs of photodiodes are placed on both sides of the motor. One pair is used to set the zero point of the rotation and the other pair is used to stop the motor. The light source, detector and spherical mirror are placed according to the optical design parameters. The optical structure is held by an L-shape aluminum plate. A heat sink is firmly attached to the surface of the light source and fixed to the plate using thermal silica gel, which stabilizes the temperature of the light source and suppresses thermal noise.

**Figure 3:**
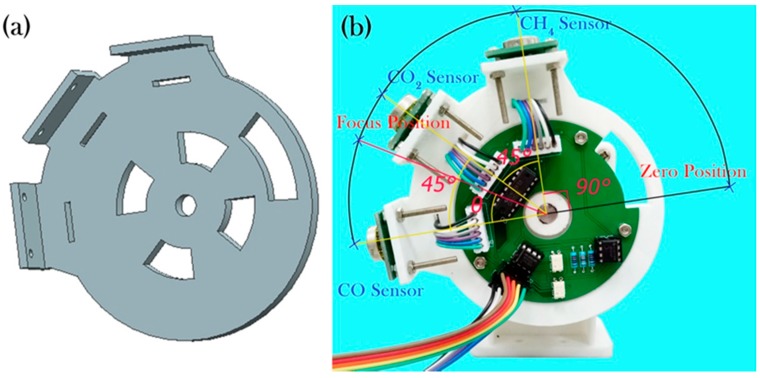
(**a**) CAD model of the disc for mounting three detectors; (**b**) Photograph of the disc with a sealed circuit board and three pyroelectric detectors for the detection of CO, CO_2_ and CH_4_.

Figure 3a shows the CAD model of the disc for fixing the three detectors. As shown in Figure 3b, the CO, CO_2_ and CH_4_ detectors were fixed with an electric circuit board. The function of the circuit is to switch the sensing channels of the detectors and to filter and pre-amplify the sensing signals. The curved hole on the disc allows the optical signal to transmit between photodiodes. The operating steps of the disc are as follows. Initially, the disc rotates clockwise until the receiver of the photodiodes acquires an optical signal through the hole in the disc. This position of the disc is considered to be the zero point of rotation. The disc rotates *θ*° clockwise so that the CO sensor reaches the focal point of the mirror (point *E* in Figure 2a) for gas detection. The angle *θ* is obtained experimentally. The sensors are arranged at an angular interval of 45° and the disc is rotated twice 45° counterclockwise to let the CO_2_ sensor and CH_4_ sensor reach the light focal point for gas detection, respectively. Finally, the disc turns clockwise back to the zero position and starts a new round of multi-gas detection.

### 2.4. Closed-Loop Rotation Control

A stepper motor, which converts an electrical pulse signal into an angular displacement, was used to control the rotation of the disc. Since the angular displacement of the stepper motor is proportional to the number of pulses and synchronized in time, the rotation angle of the motor can be precisely controlled. However, if the pulse frequency is too high, the motor cannot correctly respond to the change of a pulse, resulting in a difference between the actual position and the ideal position.

**Figure 4:**
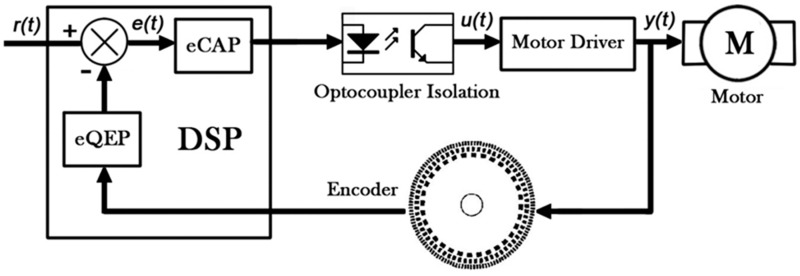
Schematic of the closed-loop control on the motor rotation.

Furthermore, as the motor switches between dynamic and static state, motor control may be more difficult to achieve due to system inertia caused by an overshoot, especially when the motor suddenly stops at high speed. If the motor rotates with low frequency, it will cause vibrations. In order to overcome these problems, a 1/8 subdivision motor driving circuit was used. The subdivision driving method results in a smooth driving current by allowing the phase current to step up to a rated value or to zero. A reduction of the current variation rate leads to a reduction of a low-frequency vibration caused by an overshoot and improves the rotation stability. The angular rotation resolution of the stepper motor was also increased from 1.8 to 0.225°. As shown in Figure 4, the angle deviation *e*(*t*) is the difference between the given angle *r*(*t*) and the feedback angle *y*(*t*). The control output *u*(*t*) is adjusted according to *e*(*t*). *y*(*t*) was measured by a photoelectric incremental encoder to form a closed-loop control. The angle value was decoded from two orthogonal square waves generated by the encoder using the eQEP module. A pulse signal was generated by the eCAP module and the DSP was also isolated from the motor by an opto-electrical coupler to increase the rotation stability.

## 3. Experimental Details and Results

### 3.1. Gas Sample Preparation

**Figure 5:**
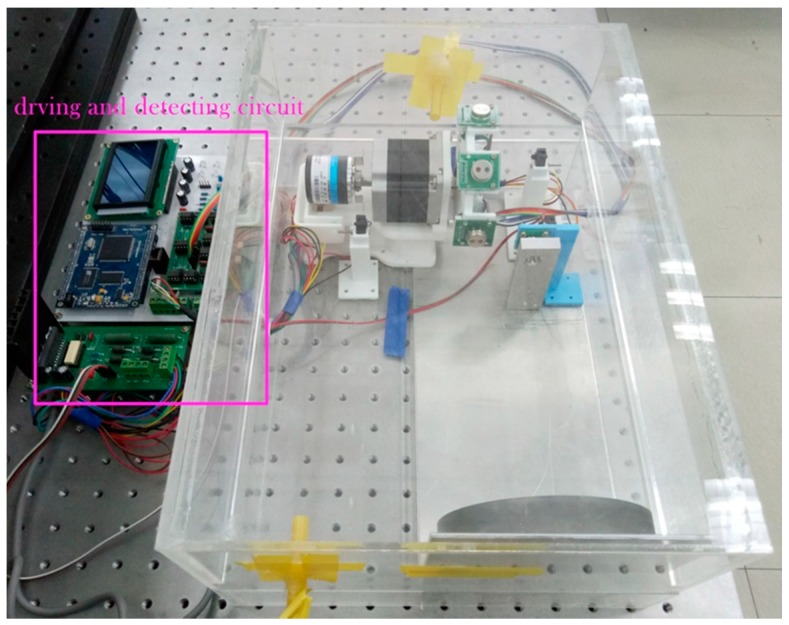
Measurement setup depicting the electrical and optical parts. The optical part was placed inside an enclosure.

Figure 5 shows the experimental setup of the multi-gas sensor, which was placed into an air-tight chamber with a volume of 15,808 mL. In order to obtain stable and accurate calibration results, a dynamic gas distribution method was used instead of a static injection distribution via a needle. Since N_2_ has no absorption in the infrared band, it was used as a carrier gas to mix with CO, CO_2_ and CH_4_ to get different gas samples for sensor calibration. It is possible to use the sensor in air for various applications, since other gases have no interference to the detection of the three target gas species. By mixing a 5000 parts per million in volume (ppmv) CO, CO_2_, CH_4_ sample with a 99.999% pure N_2_, respectively, gas samples with different concentration levels were prepared by means of two mass flow meters (Horiba Metron, S49-33/MT, Beijing, China, with a 2% uncertainty). The preparation rule can be expressed by:

(4) $$C=\frac{V_{1}\times 5000\;\mathrm{ppmv}}{V_{1}+V_{2}}$$

In Equation (4), *C* is the required gas concentration in ppmv and *V*_1_ and *V*_2_ are the required gas flow rates of the standard gas sample and pure N_2_, respectively.

### 3.2. Static Sensor Calibration

**Figure 6:**
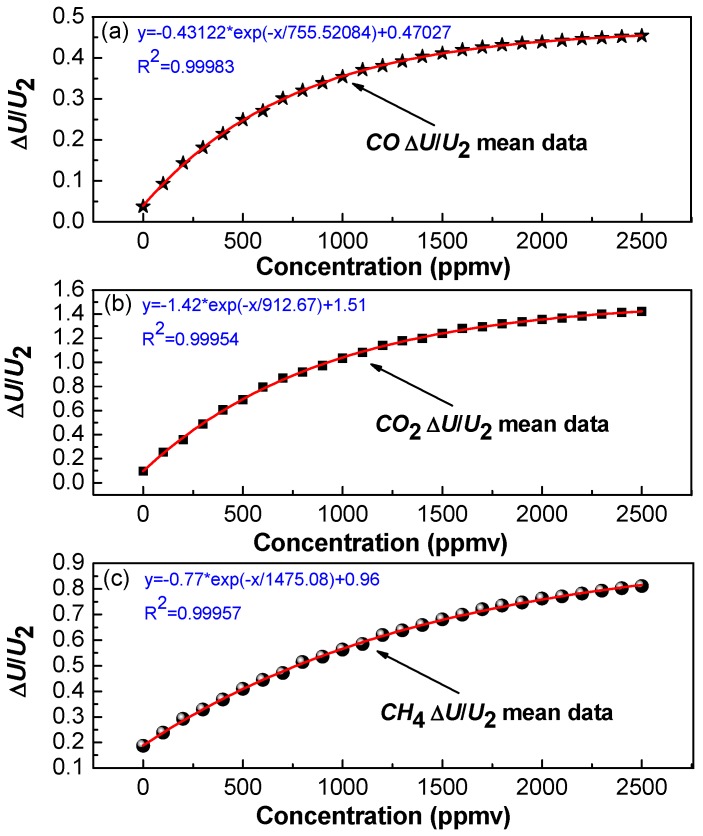
Experimental data dots and fitting curve of the ratio Δ*U*/*U*_2_ versus (**a**) CO; (**b**) CO_2_; (**c**) CH_4_ concentration.

For calibration, the motor was kept in a static state for only single-gas detection, i.e., the multi-gas sensor was being operated without rotation. Three groups of gas experiments were performed with three kinds of gas samples. In each experimental group, a series of gas samples with different concentration levels spaced apart by 100 ppmv were prepared and flushed through the chamber. The amplitude of the differential signal (Δ*U*) and the amplitude from reference channel (*U*_2_) were recorded after sensor readings were stabilized. The measurement results of Δ*U* and *U*_2_ for a time period of 8 min for each concentration were recorded. In order to eliminate the influence of noise from the light source and the detector of the detection system, we obtained a relationship between Δ*U*/*U*_2_ and gas concentration *C*. For CO, CO_2_ and CH_4_ sensing, the obtained relationship curves between the averaged Δ*U*/*U*_2_ and the concentration *C* are shown in Figure 6a–c, respectively. The fitting equations of CO, CO_2_ and CH_4_ are given by Equations (5)–(7), respectively, as

(5) $$C=-755.52\ln(\frac{0.47-\Delta U/U_{2}}{0.43})$$

(6) $$C=-912.67\ln(\frac{1.51-\Delta U/U_{2}}{1.42})$$

(7) $$C=-1475.08\ln(\frac{0.96-\Delta U/U_{2}}{0.77})$$

### 3.3. Effects of Rotation Speed on Sensor Performance

**Figure 7:**
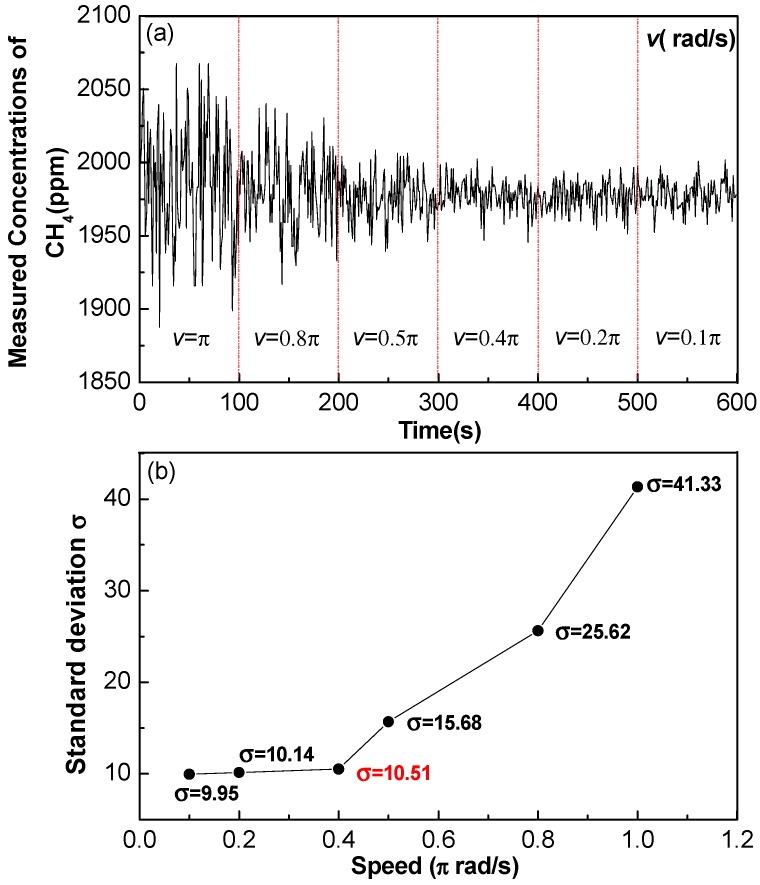
(**a**) Measured CH_4_ concentration and (**b**) the standard deviation as a function of rotation speed, where, the measurements lasted for 100 s for each speed.

Experiments were carried out to determine the impact of rotation speed on the detection stability of the multi-gas sensor operating in a dynamic state with different speed CH_4_ concentration results. Figure 7a,b show the measured CH_4_ concentration and the deviation at different rotation speeds, where for each speed, the measurements lasted for 100 s. The standard deviation continues to increase after 0.4π rad/s, which means that the closed-loop control system becomes as stable as possible, when the rotation speed is <0.4π rad/s. In order to shorten the sensor response time, the rotation speed is set to 0.4π rad/s.

### 3.4. Dynamic Response Characteristics

**Figure 8:**
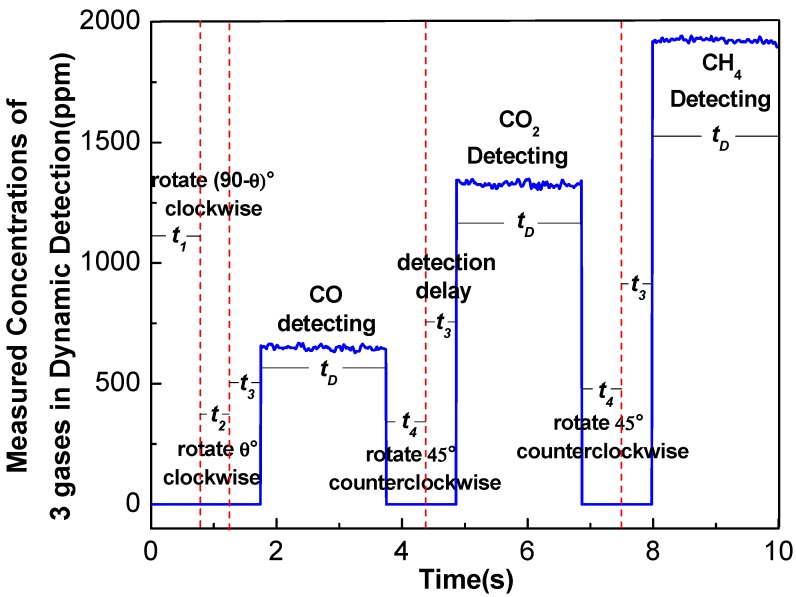
The detection of three gas species, CO, CO_2_ and CH_4_ within a period of *T* based on the multi-gas sensor system.

Figure 8 shows the working steps of the multi-gas sensor in one detection cycle. First, the disc rotates (90 − *θ*)° clockwise within a time *t*_1_ to switch the disc from the CH_4_ detection position to the zero point of rotation. Then, the disc rotates *θ*° clockwise within a time *t*_2_ to let the CO detector reach the light convergence point of the mirror. After a delay time (*t*_3_) of 0.5 s for stabilizing, the detector indicates that a measurement time (*t_D_*) of 2 s is required for signal processing and concentration determination. Finally, the disc will rotate to the CO_2_ and CH_4_ detection positions and detect the corresponding gases, respectively. As the optimal rotation speed is 0.4π rad/s and *θ* is 31°, the detection period (*T*) of the multi-gas sensor can be calculated by the following formula

(8) $$T=t_{1}+t_{2}+3t_{3}+2t_{4}+3t_{D}=(\frac{90-31}{72}+\frac{31}{72}+3\times 0.5+2\times \frac{45}{72}+3\times 2)\;s=10\;s$$

This means that the multi-gas sensor will complete the detection of three gases by scanning the detectors in 10 s with a detection time of 2 s for each gas. 

### 3.5. Comparison of Detection Stability for Dynamic and Static Operations

Initially, the chamber was flushed by N_2_ for 20 min, which was followed by injecting with a 2% uncertainty 10 mL pure CO, 20 mL pure CO_2_, and 30 mL pure CH_4_ into the enclosure. A mixing time of 20 min is required before detection. Then, the concentrations of the three gases in the chamber were calculated to be 632, 1263 and 1891 ppmv, respectively. 

**Figure 9:**
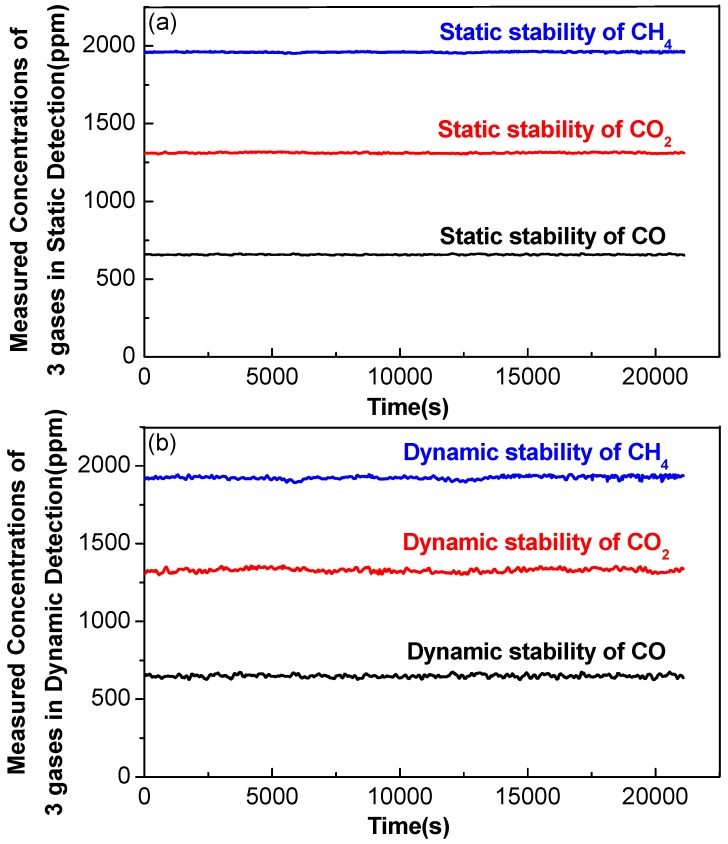
Long-term (**a**) static and (**b**) dynamic measurements of a gas mixture composed of CO, CO_2_ and CH_4_ concentration levels of 632, 1263 and 1891 ppmv, respectively.

Subsequently, the multi-gas sensor was operated in a static state without rotation. An experiment was conducted for ~5.5 h for each gas measurements and the results are shown in Figure 9a. The measured CO concentration varied from 651.47 to 664.01 ppmv with an average value of 657.55 ± 2.35 ppmv (1*σ*); the measured CO_2_ concentration varied from 1303.74 to 1318.56 ppmv, with an average value of 1311.33 ± 3.12 ppmv (1*σ*), and the measured CH_4_ concentration varied from 1948.28 to 1963.10 ppmv, with an average value of 1957.47 ± 2.98 ppmv (1*σ*).

Next, the sensor was operated in a dynamic state with rotation and ~5.5 h long measurements were performed. The results are shown in Figure 9b. The measured CO concentration varied from 625.09 to 675.26 ppmv with an average value of 649.43 ± 9.39 ppmv (1*σ*); the measured CO_2_ concentration varied from 1299.93 to 1356.26 ppmv with an average value of 1328.75 ± 11.88 ppmv (1*σ*) and the measured CH_4_ concentration varied from 1892.06 to 1943.94 ppmv with an average value of 1924.17 ± 10.39 ppmv (1*σ*).

Table 1 shows a comparison of the standard deviations between static and dynamic operations of the multi-gas sensor system. For dynamic operation, due to unavoidable motor vibrations and a rotation control error, the dynamic deviations were 3.99, 3.81, 3.49 times higher than the static deviations, respectively.

### 3.6. Allan Deviation

A long-term evaluation of the multi-gas sensor was performed in a pure N_2_ environment inside the chamber. In static detection, the sampling interval, i.e., the detection period, was set to 1 s, and in dynamic detection, the sampling interval was set to 10 s. An Allan deviation analysis was performed to study both stability and detection limit. In static detection, 3600 data points were collected for each gas in one hour to calculate the Allan variance. In the dynamic detection, 4320 data points for three gas species, i.e., 1440 data points for each gas, were collected in 4 h.

**Figure 10:**
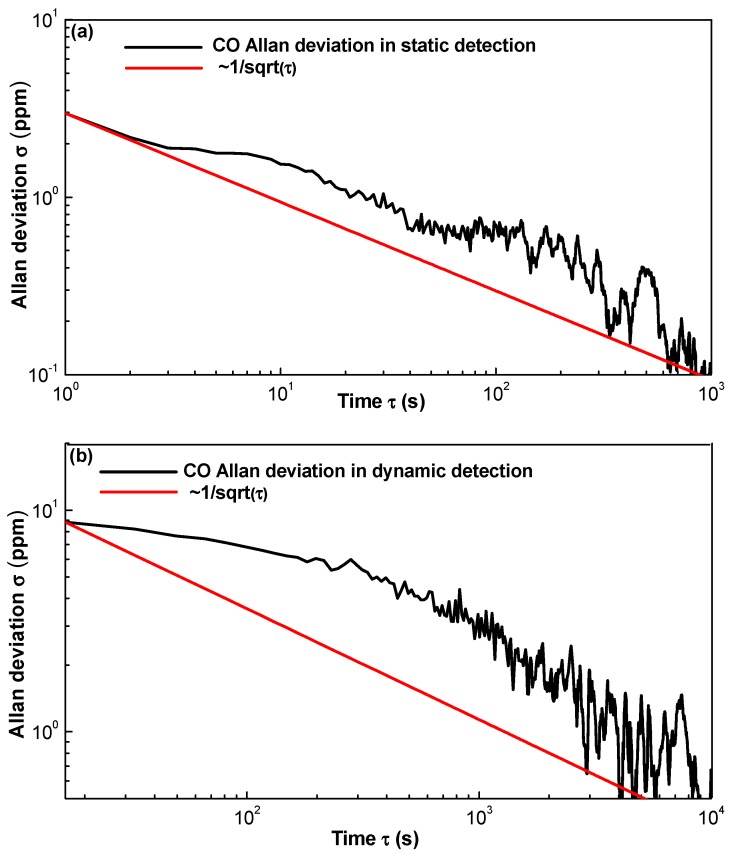
Allan deviation plot of the CO sensor in (**a**) static state with a sampling interval of 1 s and (**b**) dynamic state with a sampling interval of 10 s.

**Figure 11:**
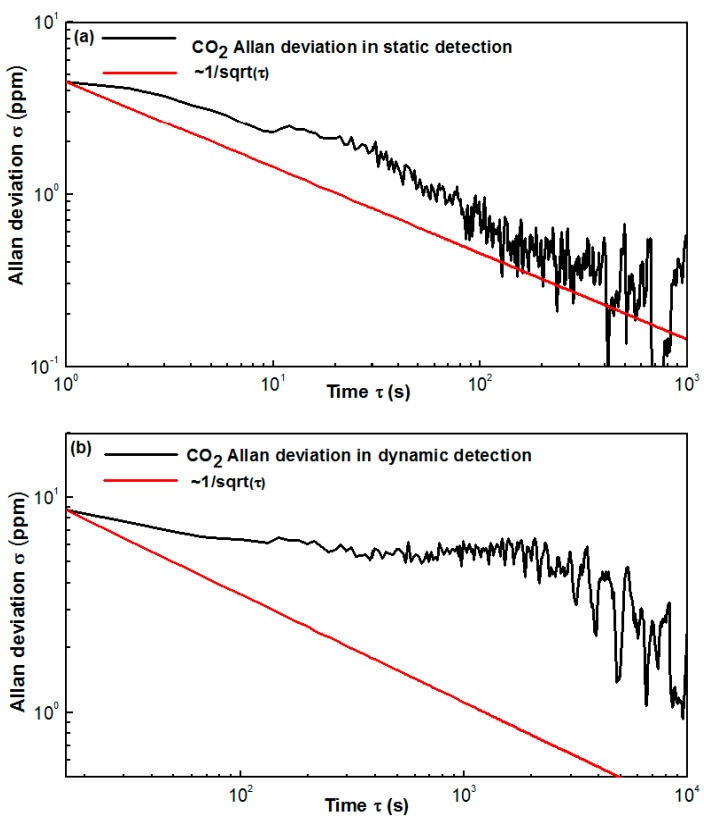
Allan deviation plot of the CO_2_ sensor in (**a**) static state with a sampling interval of 1 s and (**b**) dynamic state with a sampling interval of 10 s.

**Figure 12:**
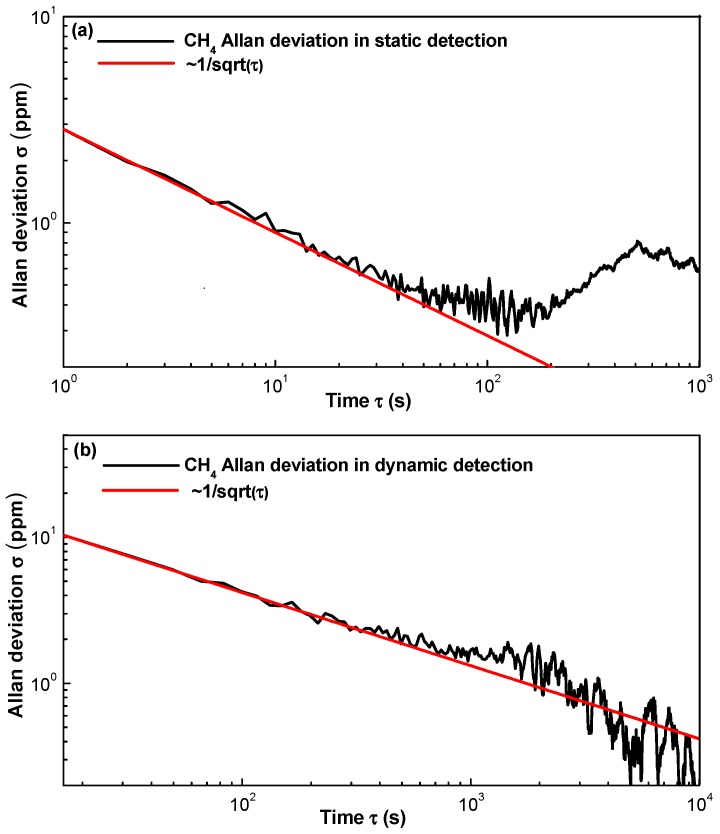
Allan deviation plot of the CH_4_ sensor in (**a**) static state with a sampling interval of 1 s and (**b**) dynamic state with a sampling interval of 10 s.

Based on Figure 10a,b, the Allan deviation for static CO detection is 2.96 ppmv with a 1-s averaging time, and that for dynamic CO detection is 8.83 ppmv with a 10-s averaging time. Based on Figure 11a,b, the Allan deviation for static CO_2_ detection is 4.54 ppmv with a 1-s averaging time and that for dynamic CO_2_ detection is 8.69 ppmv with a 10-s averaging time. Based on Figure 12a,b, the Allan deviation for static CH_4_ detection is 2.84 ppmv with a 1-s averaging time and that for dynamic CH_4_ detection is 10.29 ppmv with a 10-s averaging time. So it can be estimated that the 1*σ* detection limits under static operations are 2.96, 4.54 and 2.84 ppmv for CO, CO_2_ and CH_4_, respectively and the 1*σ* detection limits under dynamic operations are 8.83, 8.69 and 10.29 ppmv for the three gas species, respectively. The variation trend of the Allan plot indicates the noise type of the sensor system, which relates to both hardware and software. Since most of the hardware and the software for detecting the three gas species are the same, the Allan-plot difference among the three gases probably results from the differences among the three pyroelectric detectors. The stability of the newly purchased CH_4_ detector is better than the old CO and CO_2_ detectors, which may account for the observed phenomenon, i.e., a good White–Gaussian noise dominated sensor operation for CH_4_.

## 4. Conclusions

Finally, we were able to develop a multi-gas sensor system based on a single broadband light source and three pyroelectric detectors. A stepper motor driven rotation system was designed to switch to CO, CO_2_ and CH_4_ detection channels and a single-reflection spherical optical mirror was used to enhance gas absorption. A dual-channel differential detection method was used to suppress noise and improve the detection performance. Both static and dynamic measurements were performed to investigate the sensing characteristics of the sensor system. The Allan deviations for static CO, CO_2_ and CH_4_ detection are 2.96, 4.54 and 2.84 ppmv, respectively, with a 1-s averaging time and that for dynamic CO, CO_2_ and CH_4_ detection is 8.83, 8.69 and 10.29 ppmv, respectively, with a 10-s averaging time. The reported detection method has various applications such as coal mine gas detection.

## Figures and Tables

**Table 1:** Comparison of standard deviations between static and dynamic operations of the sensor system, where *σ*_s_ is the deviation in static state and *σ*_d_ is the deviation in dynamic state.

Standard Deviation	CO	CO_2_	CH_4_
*σ*_s_ (ppmv)	2.35	3.12	2.98
*σ*_d_ (ppmv)	9.39	11.88	10.39
